# Implementing a Digital Physical Activity Intervention for Older Adults: Qualitative Study

**DOI:** 10.2196/64953

**Published:** 2025-08-21

**Authors:** Cherish Boxall, Laura Dennison, Sascha Miller, Judith Joseph, Kate Morton, Jenny Corser, Joanna Kesten, Asgar Electicwala, Max J Western, Stephen Lim, Chloe Grimmett, Lucy Yardley, Katherine Bradbury

**Affiliations:** 1 Southampton Clinical Trials Unit, University of Southampton, Southampton, United Kingdom; 2 School of Psychology, University of Southampton, Building 44, Highfield Campus, Southampton, SO17 1BJ, United Kingdom, 44 02380595000; 3 School of Psychological Science, University of Bristol, Bristol, United Kingdom; 4 The National Institute for Health and Care Research Applied Research Collaboration West (NIHR ARC West), University Hospitals Bristol and Weston NHS Foundation Trust, Bristol, United Kingdom; 5 The National Institute for Health and Care Research (NIHR) Health Protection Research Unit (HPRU) in Behavioural Science and Evaluation, University of Bristol, Bristol, United Kingdom; 6 Centre for Motivation and Health Behaviour Change, Department for Health, University of Bath, Bath, United Kingdom

**Keywords:** implementation, digital technology, physical activity, physical exercise, exercising, older adult, older people, aging, mobile app, smartphone, digital health, digital intervention

## Abstract

**Background:**

Physical activity (PA) in older adults can prevent, treat, or offset symptoms and deterioration from various health conditions and help maintain independence. However, most older adults are insufficiently active. Digital interventions have the potential for high reach at low cost.

**Objective:**

This paper reports on the implementation of “Active Lives,” a digital intervention developed specifically for older adults.

**Methods:**

This study had a qualitative design. The implementation team approached a range of National Health Service, public health, community, and third-sector organizations in the United Kingdom to offer Active Lives to as large and diverse groups of older adults as possible. Alongside real-world implementation activities, research was conducted to explore what supports and inhibits the implementation of a digital intervention for PA in older adults. Data collection involved interviews with implementation partners (n=15) and the implementation team (n=3) plus extensive field notes from stakeholder communications. Inductive thematic analysis was used to analyze the data.

**Results:**

Five broad themes were developed, capturing implementation barriers and facilitators. These were (1) complex and opaque networks and influencers, (2) forming an understanding of Active Lives and its fit, (3) a landscape of competition and conflicting interests, (4) navigating unclear approval processes, and (5) shifting strategies: small and effortful to high reach and passive. Identifying key decision makers proved arduous, consuming significant time and resources, and proposals from enthusiastic implementation partners often proved impractical or overly burdensome. Health care professionals demonstrated a comprehensive understanding of the potential benefits of digital interventions in alleviating operational burdens and improving patient care. However, stakeholders from disparate sectors held reservations about digital intervention and had different views on the best approaches to supporting PA among older adults. This discord was exacerbated by conflicts with existing local initiatives, such as group exercise programs, which occasionally hindered the implementation of Active Lives. Furthermore, bureaucratic hurdles within National Health Service trust approval processes acted as formidable obstacles, dampening progress and resolve, highlighting the need for guidance in identifying sustainable and scalable practices.

**Conclusions:**

The findings highlight important implementation challenges to digital PA interventions for older adults such as bureaucratic barriers and alignment with ongoing initiatives. This research emphasizes the necessity for strategic direction and multilevel guidance to efficiently implement digital interventions for PA among community-dwelling older adults.

## Introduction

### Background

Physical activity (PA) in older adults aged 65 years and older can help prevent, treat, or offset symptoms and deterioration from a range of common health conditions such as frailty, falls, depression, cardiovascular disease, Parkinson disease, and cognitive decline [1-3]. However, in the United Kingdom, 79% of adults aged 65 years and older are insufficiently active (ie, do not undertake 150 minutes of moderate-intensity PA per week), and 32% are completely inactive (ie, undertake no moderate-intensity activity per week), with similar patterns noted in US and Dutch samples [4]. Inactivity is detrimental to older adults’ quality of life and ability to live independently, which is costly to health and social care services [3,5].

Digital behavior change interventions hold the potential to reach large portions of the older adult population. There is concern that older adults might be disadvantaged by the digital divide, where a lack of familiarity with technologies throughout their life course makes them less likely to accept, engage with, and benefit from digital solutions. In particular, older adults who are insufficiently active are more likely to experience socioeconomic disadvantage and have previously benefited less from digital PA interventions [6]. Encouragingly, the percentage of older adults using the internet has been rising, increasing the viability of digital solutions in this age group. In the United Kingdom, 83% of 65‐ to 74-year-old people were internet users, and 47% of 75-year-old people were internet users in 2019. This rose to 53% of 75-year-old people in 2020 [7,8].

### Prior Work

The “Active Lives” digital intervention is a freely available website suited to computers, tablets, and smartphones. It was developed by our research group as an inclusive, equitable, and scalable solution to help older adults increase and maintain PA [9,10]. Active Lives supports users to reduce sedentary time, increase moderate activity, and undertake strength and balance exercises. It was developed using behavior change theory in conjunction with qualitative research to ensure that the intervention was acceptable and engaging to its intended end users [10-12]. The website uses simple language and navigation to improve accessibility for people with mild cognitive impairment and lower digital or health literacy. To adapt to the heterogeneous PA abilities observed within older adults, Active Lives starts by assessing the user’s strengths and weaknesses and uses this information to tailor its recommendations, suiting it to a supportive patient-led intervention. The website addresses psychological barriers to beginning or increasing activity (eg, low motivation, low self-efficacy, concerns about injury, appropriateness, and safety), using evidence-based behavior change strategies such as self-monitoring and goal-setting [13]. These features set it apart from other available PA websites and apps [14-16]. Active Lives is currently being evaluated as a part of a 5-year multicomponent intervention (PA, diet, and cognitive exercises) to prevent cognitive impairment in older adults. To use Active Lives, users click on a website link to register with an email address and password and subsequently use the link to log in at any time. The study is registered on the United Kingdom’s clinical study registry (ISRCTN17349359), which aims to promote transparency and reduce selective reporting in interventional and noninterventional clinical studies. Preliminary findings from 2 feasibility randomized controlled trials (RCTs) conducted within primary care indicated that people randomized to Active Lives engaged well with the Active Lives intervention, increased their walking, moderate activity, vigorous activity, and strength or balance exercises compared to baseline, with no adverse events reported [9]. The intervention was co-designed with 52 older adults with and without cognitive decline to ensure acceptability. Qualitative interviews showed that the intervention was acceptable, with key facilitators of engagement identified as maintaining independence and enjoyment, while managing ill health was found to be a barrier to engagement [12].

Digital interventions such as Active Lives can potentially have huge reach at low cost, so scaling up effective interventions is a priority, but there is a research-to-practice gap, where behavioral interventions are not implemented beyond their formal evaluation in RCTs [17], and there is limited implementation-focused research to guide how this gap could be shortened [18,19]. Specifically, for navigating the unique challenges associated with implementing digital behavior change interventions [17,20,21].

Implementation research spans various stages of intervention development, including planning, feasibility testing, evaluation, and the implementation phase itself [13]. Numerous studies have been conducted during digital intervention development and integrated within feasibility testing and evaluation, providing insights into factors that may influence future scale-up [22-26]. However, there is a paucity of published research investigating implementation following successful development and evaluation.

Existing studies in this domain have primarily focused on aspects of the digital intervention itself or end-user uptake, use, and engagement [27,28]. Some research has explored implementation barriers and facilitators within the context of large-scale digital service transformation programs involving extensive consortiums [20,29].

Nevertheless, there remains a gap in the literature regarding the implementation research on scaling up a single, fully developed intervention, particularly concerning the process of engaging partner organizations to reach patients or end users. Recent commentaries have highlighted various challenges encountered when attempting to make evidence-based digital interventions freely and widely available after the RCT, such as in eczema management [30] and asthma management [21].

### The Goal of This Study

Digital interventions may be an effective solution for older adults to increase PA, but little is understood about the process of engaging with individuals and organizations that have the potential to enable reach to patients and end users. In this study, we explore the influencing factors to the implementation of a digital PA intervention aimed at older adults. We were interested in what supports or inhibits implementation and how challenges were perceived, approached, and managed from multiple perspectives, including the academic team and individuals involved in the implementation process, an approach recommended by Murray et al [31].

## Methods

### Implementation Strategy and Context

The implementation activities took place in the United Kingdom between September 2020 and January 2023. In this paper, the terms “implementation team” and “implementation partners” are used to describe 2 broad sets of actors.

The implementation team was academics trying to get Active Lives to end users (via implementation partners). The implementation team was led by coauthor KB, a health psychologist involved in developing and evaluating Active Lives and supported by 2 postdoctoral researchers (co-authors JJ and SM).

Implementation partners were people, including clinical and academic grant coapplicants, who were approached about facilitating the implementation of Active Lives either at a strategic or practical level. The term is used in this paper to capture all such individuals, regardless of whether they eventually implemented Active Lives. Due to the location of the implementation team and their professional network, most of the implementation partners were based in the south of the United Kingdom. We began implementing Active Lives in the most socioeconomically deprived areas within Southampton and Portsmouth. These regions were selected due to their high prevalence of low education levels and established connections with local charities that serve vulnerable populations. Following these initial areas, Active Lives experienced organic expansion in a diverse range of health care and community organizations based in Devon, Berkshire, Sussex, Reading, Isle of Wight, and Dorset.

Active Lives is designed to be used without specialist support or follow-up. Therefore, the key action necessary to roll out Active Lives was that implementation partners shared a link (ie, a URL) with older adults and a brief verbal or written explanation of Active Lives, ideally framed as an endorsement or recommendation to encourage self-management of PA. The implementation team was open to exploring implementation opportunities that emerged naturally during the project. Several routes were anticipated as starting points through insights from pilot work and planning discussions at the grant proposal stage, including NHS falls and physiotherapy services, public health, social prescribers, charities, and community organizations.

The implementation team planned a theoretically informed implementation strategy. A thorough account of this is beyond the aims and scope of this paper. Table 1 outlines how 3 key theories and frameworks [32-34] were referred to for implementation planning and data collection activities. As implementation progressed, a Plan Do Study Act approach [35] was used to review and adapt the implementation strategy and activities.

**Table 1.** Theories and models used to plan the Active Lives implementation strategy.

Theory or model or framework	Overview of how the theory or model or framework was used	Examples
RE-AIM^a^ [32]	RE-AIM was used as a broad overarching framework to consider important aspects of intervention success. It helped us to consider key implementation activities and to plan data collection (logging implementation activities and digital intervention use data).	RE-AIM guided decisions on prioritizing routes that were likely to have high Reach. For example, more of the implementation team’s limited time was invested in attempts to get implementation partners to set up routine digital or paper-based communication from NHS^b^ services that serve thousands of people rather than ad-hoc face-to-face referrals via volunteers in small local community organizations that serve hundreds of people. RE-AIM also helped to identify types of data that needed to be collected for evaluation purposes (where feasible). For example, the decision was made to create bespoke Active Lives URLs for different organizations and routes, so that users signing up via different routes could be tracked separately (Reach).
Normalization Process Theory (NPT) [33]	NPT was used to anticipate the work that implementation partners would need to undertake in order to make Active Lives become sustainable, normal practice within their organization. It led us to consider how implementation plans and activities could facilitate these processes. It also influenced data collection (topic guides).	When considering the coherence concept, the implementation team developed a set of resources (videos, PDFs, and presentations or pitches) to facilitate implementation partners “sense-making” around Active Lives, particularly drawing out its unique features and making clear how it would be used. This was iteratively tailored to different settings based on the implementation team’s evolving understandings of the context and priorities of each implementation partner.
The Consolidated Framework for Implementation Research (CFIR) [34]	The CFIR was used to identify, pre-empt, and plan for how to respond to a wide range of barriers and facilitators to implementation. It also influenced data collection (topic guides).	The outer setting domain: External pressure construct focused the implementation team on the need to be aware of contemporary pressures on implementation partners and how this would link to their interest in and ability to implement Active Lives. For example, within the NHS, relevant factors included post–COVID-19 hospital waiting lists (and the “Waiting Well” agenda), deconditioning in older adults, various new strategies and policies, and restructuring of teams and services. Some of these “external pressures” aligned well with Active Lives, while others indicated competing pressures for potential implementation. The inner setting domain: Internal pressure construct highlighted the need to understand (in advance) the organizations and teams that were potential implementation partners. Constructs such as “culture,” “tension for change,” and “relative priority” helped to plan and tailor communications and sometimes aided decision-making about spending time pursuing routes that were not working.

a RE-AIM: Reach, Effectiveness, Adoption, Implementation, Maintenance.

b NHS: National Health Service.

### Participants

Health care professionals (HCPs; n=approximately 200), health service managers, public health specialists, sports and PA experts, and community and charity workers were involved in emails, meetings, or discussions about implementing Active Lives. We sent an invitation email to those who represented or had influential power in a group who were approached regarding the implementation of Active Lives, irrespective of their progress. The academic members of the implementation team conducting much of the work with implementation partners were also interviewed.

### Data Collection

#### Overview

After completion of consent forms, guided semistructured interviews (SuppMat3 and 4) were completed using videoconferencing software (Microsoft Teams) and were audio-recorded and transcribed verbatim. The personnel who conducted the interviews were not directly involved in implementation activities and consisted of a Predoctoral Fellow who completed most implementation partner interviews and 2 experienced qualitative researchers, one of whom conducted the interviews with the academic team.

#### Implementation Team

Semistructured interviews captured the experiences and perceptions of the academic team involved in the implementation activities, including perceived barriers and facilitators (Multimedia Appendix 1).

#### Implementation Partners

Our sample size was determined pragmatically by the willingness of our partners to participate. Assessment of information power revealed a high degree, supported by the narrow aim, dense specificity, application of theory, and strong dialogue [36]. The topic guides (Multimedia Appendix 2) focused on their involvement with and perspective on Active Lives, including obstacles, facilitators, and learning points relating to implementation.

### Field Notes

The implementation team made field notes. Typically, these were reflections on meetings or other communications with implementation partners. These included perceptions of power dynamics and behavior within meetings, issues that had been mentioned informally by implementation partners, personal feelings about the work involved with implementation, as well as problem-solving and suggestions for next steps.

### Data Analysis

Analysis was led by LD and was broadly aligned with a reflexive thematic analysis approach [37]. It involved inductively developing themes that capture shared meaning and creating interpretations and narratives around observations and patterns recognized within the dataset. Analysis began with familiarization (listening to audio recordings and reading transcripts and field notes). NVivo (QSR International; version 12) was used to code the data related to the research question. Codes were organized and clustered into preliminary themes, which captured important features and patterns within the data. Themes were iteratively reworked and relabeled, with ongoing discussion from coauthors, to add additional analytic perspectives and ensure that the final themes were insightful, coherent, and well-supported by the data.

### Reflexivity

Consistent with reflexive thematic analysis, we consider our analysis and the themes we generated to be inextricably linked to our own experiences, assumptions, and positionalities [37]. An important issue to draw attention to is the multiple and overlapping roles of coauthors KB, JJ, and SM who were involved in the development of Active Lives and formed the implementation team (and as such were also interview participants and producers of field notes). Although LD did not participate in implementation meetings and activities, her academic background, experiences, loyalties, and goals are naturally more aligned with the implementation team than the implementation partners.

### Research Design and Reporting

The study is reported using the StaRI (Standard for Reporting Implementation Studies) statement for implementation studies [38] and the COREQ (Consolidated Criteria for Reporting Qualitative Research) checklist for qualitative studies [39] (Checklists1  2).

### Ethical Considerations

Ethics approval for this research was obtained via Health Research Authority and Health and Care Research Wales (ref 22/HRA/0130) and from the University of Southampton (ref 57536.A2). All participants were given an information sheet outlining the study’s objectives and provided informed written consent. Participants were informed prior to and at the time of interview that their involvement was voluntary. To ensure anonymity and confidentiality, participant data were replaced with a numerical code (eg, 1001001), and identifying information within the transcript was anonymized prior to data analysis.

## Results

In total, 17 interviews were carried out.

### Implementation Team

Three interviews were with members of the implementation team. Interview durations varied between 76 and 97 minutes.

### Implementation Partners

A total of 22 implementation partners were invited for interviews from 12 different organizations. In total, 8 people declined the invitation to interview, and 1 did not reply. In total, 14 interviews were completed with 13 implementation partners. This included NHS HCPs, most of whom were in senior or strategic leadership roles, partners from an Academic Health Sciences Network (AHSN), employees from sports or PA organizations, and from charities or community groups serving older adults.

Due to a change in roles and organization during the study, 1 interview was repeated because the participant was able to share implementation perspectives from both a strategic (regional) and organizational (health care department) level.

Interviews with implementation partners lasted between 27 and 57 minutes (median 33, IQR 30.5-47 minutes). Detailed demographic information is withheld from reporting in the paper to ensure the anonymity and confidentiality of implementation partners’ participants.

### Overview

Five broad themes were developed from the data, as depicted in Table 2. Below, each theme is explained, and supporting quotations are presented. To preserve anonymity, names and organizations are replaced with participant ID numbers and tagged as either implementation team (eg, ImpTeam_1) or implementation partner (eg, ImpPartner_2). For the latter, a broad indication of their professional role or sector is given (eg, NHS HCP).

**Table 2.** Themes and subthemes.

Theme	Subthemes
Complex and opaque networks and influencers: This theme is about the complexities of identifying people with sufficient interest and power to help implement Active Lives.	Identifying open doors and gatekeepers: This subtheme is about the difficulties in identifying suitable implementation partners. Champions and “antichampions”: This subtheme is about the people whose actions facilitated and blocked implementation.
Forming an understanding of Active Lives and its fit: This theme is about how implementation partners understood (or misunderstood), labeled, and appraised Active Lives and grasped how it might fit into their organizations and alongside their priorities. It also covers how the implementation team facilitated sense-making about Active Lives.	Labeling Active Lives as a good thing: This subtheme is about the positive aspects of Active Lives that implementation partners recognized. Having doubts and concerns about Active Lives: This subtheme relates to objections to and worries about Active Lives that Implementation Partners had.
A landscape of competition and conflicting interests: This theme covers how Active Lives implementation occurred in a context of competition with other interventions and initiatives and how this inhibited or blocked implementation	—^b^
Navigating unclear approval processes: This theme is about how a lack of clear process for large organizations (particularly NHS^a^) to approve Active Lives or sign off on decisions created burden and uncertainty, slowing or blocking implementation.	—
Shifting strategies: small and effortful to high reach and passive: This theme is about how implementation partners were initially attracted to small-scale, effortful routes but could be persuaded to find more efficient, scalable routes.	—

a NHS: National Health Service

b Not applicable

### Complex and Opaque Networks and Influencers

#### Identifying Open Doors and Gatekeepers

The implementation team’s professional networks made connections and introductions to potential implementation partners and helped them to understand “the landscape [...], the groups that are influential” (ImpTeam1). Implementation partners in both NHS and community settings considered supporting older adults to become more active to be relevant to their job, but it was rarely a key responsibility.

*[It’s] a “side thing” for most people.*

[ImpTeam3]

Language reflecting the need for openness was used frequently; pursuing “open doors” (ImpPartner1_AHSN) rather than “pushing against closed doors” (ImpPartner2_AHSN) or “trying to knock [the door] down” (ImpPartner9_sports/PA sector). However, determining the openness of “doors” was unpredictable despite steers from a wide network. Many initial meetings were unproductive. Others seemed positive; yet, further contact or action never materialized. “Gatekeepers” (ImpTeam1) were gradually uncovered; people were enthusiastic but also, crucially, held decision-making power.

*if they’re not the right decision-maker then it’s quite-it’s a dead end.*

[ImpTeam1]

Difficulties (highlighted below) in recognizing open doors and gatekeepers at the project outset meant “traction” (ImpTeam1) was slow, and progress only began “snowballing” (ImpTeam2), as project funding was running out.

#### Champions and Antichampions

At multiple levels (eg, regional to local department), champions were seen as crucial for spreading Active Lives and were recognized as “adding massive credibility” (ImpTeam1) and garnering and sustaining interest, commitment, and action.

*[I’m] kind of banging away at the drum.*

[ImpPartner8_NHS-HCP-leadership]

*I have encouraged others to use it in their clinical work. I have just made sure that there weren’t any barriers locally.*

[ImpPartner10_NHS-HCP-leadership]

People who championed Active Lives or facilitated introductions noted professional benefits, including prestige, improved networks, and better ability to perform their jobs. However, the work needed to support implementation could also be burdensome (see Forming an Understanding of Active Lives and Its Fit section).

Besides champions, participants discussed people who deliberately blocked or inhibited implementation: “Anti-Champions” (ImpTeam1). Sometimes blocking was overt, directly speaking out against Active Lives or behaving in rude and hostile ways. Participants also suspected that antichampions who were hostile to Active Lives were obstructing progress from behind the scenes. Considering this, ImpPartner10_NHS HCP-leadership advised of the need to “go around and above” those with a reputation for “territory defending” (Fieldnote-ImpTeam1). Although participants expressed some sense of mystery about the dynamics at play, antichampionship tended to be interpreted as arising from misunderstanding Active Lives or having competing interests (see Forming an Understanding of Active Lives and Its Fit section).

### Forming an Understanding of Active Lives and Its Fit

#### Labeling Active Lives as a Good Thing

All implementation partners recognized PA in older people as a magic medicine (ImpPartner1_AHSN). They appreciated the need to better support PA in community and clinical settings.

An absolute no-brainer in having a benefit.

[ImpPartner15_NHS HCP]

Most viewed Active Lives enthusiastically, valuing its coproduced development with older adults and its tailored activity recommendations designed to accommodate the population’s diverse abilities, including those with chronic health conditions and lower levels of digital literacy. Coverage of psychological barriers to PA, support with different types of PA (including strength or balance), and emphasis on activity within daily life were appreciated. The absence of cost was important. The trial evidence base was valued by HCPs, whereas other partners found case studies and testimonials more persuasive.

Active Lives was perceived as having potential for extensive reach and broad relevance. One HCP declared that the patient group she would like to use it with was “basically the world!” (Fieldnote-ImpTeam2), another felt that “it could fit for anybody” (ImpPartner14_NHS HCP). Nevertheless, some participants described how PA interventions struggle to fit within the NHS, which is organized around conditions, not prevention.

There’s just no agenda for prevention within the NHS or proactive interventions.

[ImpPartner10_NHS HCP-leadership]

[the broad appeal] is partly its downfall [...] if you can be quite specific about where it fits and what pathway and what condition it fits with, you can almost be quite prescriptive in its implementation.

[ImpPartner7_NHS HCP]

The implementation team, mindful of this, developed skills in understanding services, recognizing unmet needs, and helping HCPs to identify exactly how Active Lives fit: “the value to the organisation, the individual” (ImpPartner2_AHSN). They gradually honed the way they articulated the offer, finding the right “hook” (ImpPartner1_AHSN). When approached in this way, HCPs were particularly positive, easily grasping how Active Lives would benefit their patients and their working practices.

I immediately knew that it would fit in to my current work.

[ImpPartner10_NHS HCP-leadership]

Somewhere easy to signpost people to.

[ImpPartnerP12_NHS HCP]

Active Lives’ value was readily recognized when services were undergoing new challenges or change. For example, when new regional or department-level plans were introduced. Its value was readily seen in the context of excessive waiting lists, deconditioning of older people, and hospital pressures resulting from long hospital stays.

[It] ticked all the boxes etc ... particularly during and since COVID.

[ImpPartner2_AHSN]

#### Having Doubts and Concerns About Active Lives

Most implementation partners, particularly within the NHS, considered Active Lives’ digital format suitable for many older adults. However, the suitability was occasionally rejected or questioned, particularly by those in community organizations serving deprived areas. Digital exclusion seemed to drive concerns. ImpPartner11_Charity/community sector described “grinding poverty” and “language barriers” in her deprived urban area. There were *“*just–too many barriers ... it’s not to say that it’s not a fantastic thing.” Another described how

Around 60% of [the older adults we serve] are either not digitally included at all or their activity would be very restricted.

[ImpPartner4_Charity/community sector]

Several implementation partners were highly concerned that digital interventions could exacerbate isolation and loneliness.

*I would never ever support anything that would completely remove human contact.*

[ImpPartner11_Charity/community sector]

There was also a broader concern about digital services threatening the existence of face-to-face services. One implementation partner described older adults being reluctant to engage with (non-PA) digital services “because they feared that if they started engaging with us digitally, we’d decrease their access to face-to-face stuff” (ImpPartner4_charity/community sector).

Implementation partners consistently communicated that Active Lives was only suitable as part of a broader PA offer and repeated the importance of ensuring choice for patients or users.

As part of a suite of interventions.

[ImpPartner10_NHS HCP-leadership]

A menu of solutions [...] a pick and mix.

[ImpPartner1_AHSN]

Beneficial to add to a selection of tools.

[ImpPartner6_NHS HCP]

The need to offer older adults a variety of PA approaches was acknowledged by the implementation team. Nonetheless, some implementation partners believed that Active Lives sought to replace rather than complement other services (see A Landscape of Competition and Conflicting Interests section).

Occasionally, concern was expressed over the safety of digital interventions for PA in older adults, including about dizziness or falling if PA was unsupervised and potential litigation if someone was injured.

[The public health team thought] services that are not provided by a certified exercise specialist might be risky.

[ImpTeam1]

Another unfavorable perception of Active Lives was linked to a distrust of academics and a view that researchers are detached from practical realities, thereby rendering evidence-based interventions inappropriate to meet the local population’s needs. Importantly, this disconnect with academia seemed limited to community organizations who prioritized local insights to develop and commission PA services that got people out of the home and socializing, a singular approach that was privately challenged by the implementation team.

*They trust each other and they don’t necessarily trust academics.*

[ImpTeam1]

One implementation partner described disinclination to implement Active Lives, which they considered “an academic research piece” rather than a *“*practical intervention that is really looking to make sustainable change in local communities” (ImpPartner3_Sports/PA sector).

### A Landscape of Competition and Conflicting Interests

Most data underpinning this theme came from the implementation team interviews and their field notes about meetings and conversations. A key barrier to initial buy-in and sustained action on implementing Active Lives was a dedication to existing PA initiatives adopted by services and organizations. Specifically, Active Lives was perceived as posing a threat to and being in competition with other ventures.

A common pattern of concerns raised by implementation partners from the PA sector related to how they believed digital provision threatened or competed with doing PA outside of the home. A specific concern was that Active Lives would stop people from attending group exercise programs that they had commissioned. This appeared to be based on a misunderstanding that Active Lives motivated the use of exercise videos to follow alone at home rather than being a motivational and supportive behavioral intervention to encourage older people to adopt whatever form of PA they preferred (including group activities outside the home). The implementation team tried to address this, articulating how Active Lives could complement group exercise provision, serving different types of people, or potentially providing a “stepping stone” (ImpTeam1) to support currently inactive people to increase confidence and motivation to attend classes.

[we learned to] position Active Lives so that it didn’t seem a threat ... So they could see that this was complementary, rather than alternative.

[ImpTeam1]

Perceived conflict and competition were also apparent with condition-focused self-management digital interventions with a PA component (eg, diabetes and chronic obstructive pulmonary disease), informational websites about PA, and national PA promotion campaigns.

[physical activity sector partners] felt a little threatened by it, because they felt that that was the area that they were trying to involve themselves in as well. I think there was a bit of a perceived conflict there.

[ImpPartner5_NHS HCP]

They’re already paying for [disease self-management DI]; they don’t want to confuse it by getting something else even though he thinks [Active Lives] is better.

[ImpTeam2]

Some of the commitment to other approaches and unwillingness to consider Active Lives seemed to stem from personal involvement with development or commissioning (eg, where a service had created its digital resources).

He’s already got his own thing that he’s doing and he doesn’t want to promote [Active Lives].

[ImpTeam2]

The implementation team described experiencing territorial and defensive behavior, “[an Implementation Partner said] I was stepping on toes” (ImpTeam1). A sense of territory was reflected in the language of implementation partners, such as “hunting grounds” (ImpPart1_AHSN).

Other reasons for loyalty to existing interventions also became clear. With one charity, it emerged that a group PA program was generating income, and a successful adoption of Active Lives was perceived as threatening this income.

[that the income stream] was more of a motivator for them than us saying “here’s something that can meet the need.”

[ImpPartner1_AHSN]

Furthermore, informal conversations with some implementation partners highlighted fear of redundancy; digital interventions threatened the viability of in-person services and job security. The perception of an overcrowded and competitive space was frustrating to the implementation team who could differentiate Active Lives’ unique scope and features and relevance to different populations.

It’s something completely different!

[ImpTeam1]

Conflicting interests were often undeclared in formal meetings and discussed off-record. Sometimes it only became apparent to implementation partners when they consulted more widely.

The concerns expressed within this theme meant that several charity sector PA partners chose not to take on Active Lives. While there were clear financial concerns from these partners, relating to the perception that Active Lives could reduce attendance at their in-person exercise classes, we also examined the funding sources of these services to understand whether funding might be related to interest in rolling out Active Lives. Charities that were approached to implement Active Lives were usually funded by a mix of funding streams, which included donations, county council support, and funding from the national lottery and Sport England. Those most reluctant to participate were more likely to be part-funded by Sport England and provided their own in-person PA classes. However, once we had understood the concerns of these organizations, we were able to refine our pitch to more clearly demonstrate how Active Lives could help motivate more people to attend their in-person classes rather than competing as an alternative to in-person support. After this, when we approached other similar organizations who were part-funded by Sport England (in some cases, part of the same organization in a different area), those services often did decide to roll out Active Lives.

### Shifting Strategies: Small and Effortful to High Reach and Passive

The people who most recognized the value of Active Lives tended to be busy NHS HCPs who had limited “headspace” (ImpPartner5_NHS HCP) and expressed frustration with their ever-expanding workload and responsibilities. Typically, implementation partners initially suggested ways they wanted to spread Active Lives within their service organizations. They often proposed routes that were likely to be low reach, extremely effortful, or both.

People can often come up with ideas that aren’t going to work.

[ImpTeam1]

Low-reach suggestions included waiting room posters or mentioning Active Lives on a long list of unrelated resources or a low-traffic website, strategies the implementation team was not eager to pursue.

If we’re in a list, we just get buried.

[ImpTeam3]

Enthusiastic implementation partners usually felt that in-person signposting to patients or service users was the ideal way to provide access to Active Lives.

*Signposting from kind of healthcare professionals is the kind of optimum route.*

[ImpPartner2_AHSN]

HCPs took the suitability of Active Lives for their patients very seriously and wanted to ensure that Active Lives was discussed with the right patient at the right time to ensure safety and appropriateness. HCPs described needing to be both familiar with and in agreement with the content of Active Lives before discussing with a patient.

We’ve basically both just signed up to it and had a look through ourselves.

[ImpPartner13_NHS HCP]

If it goes wrong, and someone doesn’t like the outcome of it, or doesn’t enjoy it, or thinks that it’s going to cause them an issue, it’s us that the blame will fall back on.

[ImpPartner6_NHS HCP]

Some implementation partners anticipated it being necessary to verbally “onboard” users and even demonstrate how to use Active Lives.

Click on this, this is what you’ll see. This is how you’ll interact with it, this is what you’ll do.

[ImpPartner7_NHS HCP]

However, implementation partners also highlighted how unfeasible this would be in the context of being overloaded and unable to cope with the “creep” (ImpPartner5_NHS HCP) of requirements for each clinical consultation. Having multiple HCPs within services absorb and retain information about Active Lives and finding time to discuss it with patients also proved unrealistic to organize and sustain.

To try and remind [colleagues], encourage them, to offer it as an option [...]. It can become quite overwhelming.

[ImpPartner6_NHS HCP]

The implementation team was clear that this was not a sustainable way to fulfill “the promise of digital—that you can reach lots and lots of people” (ImpTeam1).

Recognizing the tendency for implementation partners to identify overly effortful or low-reach routes, the implementation team began to offer keen implementation partners a consultation meeting and follow-up support to pursue routes “that are more passive, where people are not relied on” (ImpTeam1). Successful strategies included embedding information about Active Lives within routine letters or digital communications to patients (eg, SMS text messages) or otherwise signposting Active Lives into an existing process or protocol. Once suggested, these routes were enthusiastically supported, especially when examples of other organizations that were already doing this were given.

### Navigating Unclear, Burdensome Approval Processes

Implementation partners from various settings expressed caution around implementing digital interventions, uncertainty about how to assess them, and a lack of time to learn. Some highlighted how they and their colleagues were not confident with digital technologies: “we’re all a bit out of date” (ImpPartner15_NHS HCP).

In this context, NHS HCPs faced extra work if they tried to gain organizational approval to implement Active Lives.

You think, “this might be hard work.” It almost puts a break on before you go any further.

[ImpPartner15_NHS HCP]

The work usually involved multiple meetings as discussions moved “up the food chain” (ImpTeam1). The process was extremely slow and frustrating to navigate for both implementation partners and the implementation team.

Feels like wading through mud.

[ImpPartner8_NHS HCP-leadership]

HCPs found themselves burdened with complicated approval paperwork and were expected to attend meetings about information governance. One HCP (too busy to be interviewed) described it as “overwhelming and intimidating” and “heavy handed” (Field notes-ImpTeam2). Uncertainty characterized the approval process, as different NHS trusts and services were inconsistent, and the process was not publicized. The implementation team felt unable to help implementation partners when discussions were ongoing within trusts because they could neither see what was happening nor anticipate what objections might arise.

[it is like] a corridor with ten doors on it and you don’t even know what those doors are, who they lead to.

[ImpTeam2]

During the implementation project, implementation partners encountered crises including the COVID-19 pandemic, industrial action, and NHS trust “critical incidents.” An easy, successful implementation route came about during the chaos of the COVID-19 pandemic when an HCP added the Active Lives URL and a sentence explaining its benefits to a routine patient letter, without the typical wider consultation.

*We perhaps could have gone further up and asked for permission at a higher level ... they might have said no, and I think the reason that I say that is they might’ve said no simply because they don’t have time to give it due consideration*.

[ImpPartner5_NHS HCP]

## Discussion

### Key Findings and Implications

This study captures the process of engaging with individuals and public sectors who have the potential to implement “Active Lives,” a digital PA intervention for older adults. The emergence of previously unanticipated concerns not documented in existing literature offers novel insights into the challenges and opportunities associated with implementing digital PA interventions for older adults.

One prominent challenge highlighted in our study was the difficulty in identifying suitable implementation partners. Despite an extensive network available to facilitate introductions, finding “open doors,” gaining access to decision makers, and advancing discussions about implementation needed considerable time investment spanning several months each time a new person was introduced. Although the academic team was able to communicate the underpinning principles and unique aspects of the intervention and sense-making about how the intervention matched patient or user needs, the time required to engage with multiple partners was incompatible with workloads and incentivization within academia, as noted in previous reports, where academics have attempted to implement digital interventions [21,30].

Furthermore, the divergence in perspectives between HCPs and stakeholders from the community, public health, and PA sectors accentuates the need to accommodate diverse organizational and individual needs and preferences. While HCPs valued the evidence base and viewed Active Lives as a valuable tool for alleviating operational pressures and improving patient health, other sectors relied on local insights and were resistant to shifting beliefs about getting people out of the house and socializing as an optimal strategy for older adults.

Additionally, a competitive landscape for PA interventions added further complexity to bridging gaps in perspectives of Active Lives. The presence of a crowded market in PA support provision was also unanticipated and undocumented in the literature, beyond an overall awareness that people and organizations may have conflicting priorities, agendas, and goals [26]. This study identified how revenue streams from in-person group classes could tie organizations to in-person approaches and how job insecurity and personal involvement with developing and commissioning other PA services might inhibit the uptake of digital interventions. This study also showed how certain individuals and groups seemed to act as significant blocks to implementation (antichampions) because their links to multiple organizations made their influence far-reaching.

A recent review of implementation studies of PA interventions in community settings revealed multiple barriers and facilitators relating to intervention characteristics, setting, individual characteristics, and processes of implementation [18]. However, none of the included studies were digital interventions, and only 1 targeted older adults.

Implementation theories and frameworks highlight the importance of the people involved with the implementation and their influence, attitudes, and behaviors [19,33,34]. Reviews of implementation studies of PA interventions in community settings recommended paying more attention to how individuals affect implementation [18]. However, at the project outset, it was unclear who would be involved, where champions and antichampions would be found, and what their objections to Active Lives would be.

Academic teams leading implementation efforts may not be the best route to scaling digital interventions. Commercialization, with a dedicated team to support initial and sustained implementation work, could be a more feasible route to widespread implementation and scaling up. This is problematic, however, as the intervention being cost-free to both services and end users was an important implementation facilitator.

The principle of considering the implementor’s perspectives on suitable local ways of implementing interventions is highlighted within various implementation frameworks [18,34]. However, this study suggests the need to anticipate implementation partners’ inclination toward low-reach or overly complex routes and discourage such approaches to maximize the benefits of digital interventions reaching many users at a low cost. Working collaboratively while guiding toward alternatives that are scalable and realistic is important.

However, despite ostensibly straightforward routes, the unpredictable and bureaucratic approval systems for digital interventions within the NHS were a substantial burden, potentially limiting adoption to only the most committed implementation partners. A consistent, proportionate approach is needed within NHS trusts to support HCPs in adopting evidence-based digital interventions that align with clinical and organizational needs. We did, however, identify that champions of Active Lives were motivated by professional benefits from involvement such as prestige and improved networks, a finding also noted elsewhere [18]. Emphasizing these benefits when engaging implementation partners in future projects may help offset their frustrations with the work involved.

Finally, future implementation strategies might need to consider how digital PA interventions can be used as stand-alone or complementary interventions that can be used alongside existing in-person services. This approach may also overcome concerns regarding equitable access and engagement with PA, specifically in deprived areas.

### Strengths and Limitations

This study identified several critical challenges in implementing a digital PA intervention for older adults. Key findings include the time-intensive process of identifying suitable implementation partners, divergent perspectives between HCPs and community sector stakeholders, an unexpectedly competitive landscape for PA interventions, the presence of influential “antichampions,” a tendency among partners to suggest low-reach implementation strategies, burdensome approval processes within health care systems, and the incompatibility of engagement timelines with academic workloads. The study also revealed potential facilitators, such as the professional benefits for implementation champions and the incentive of the intervention being cost-free. These findings underscore the complex interplay of organizational, individual, and systemic factors influencing the implementation of digital health interventions, highlighting the need for tailored, multifaceted implementation strategies that address diverse stakeholder concerns and leverage existing organizational structures.

This study’s strengths lie in its in-depth qualitative approach, using reflexive thematic analysis of field notes and interviews to explore real-world implementation dynamics. Our research contributes to the relatively underexplored area of implementing digital PA interventions for older adults.

However, several limitations warrant consideration. The brief duration of interviews with implementation partners and potential recruitment bias toward supportive individuals may have constrained the breadth and depth of insights. Furthermore, the focus on early implementation stages and prominent stakeholders limits the generalizability of findings to sustained implementation efforts.

Project funding timescales necessitated conducting interviews early in the implementation process, primarily focusing on key introducers, gatekeepers, and champions. Consequently, the analysis primarily offers insights into implementation partners’ decision-making processes regarding Active Lives adoption rather than the ongoing implementation “work” [33]. Additionally, quantitative analysis of uptake, use, engagement, and changes in PA was beyond this study’s scope.

Future research could address these limitations by incorporating longitudinal perspectives and quantitative analysis to further elucidate uptake, use, and engagement with digital PA interventions among older adults. Additionally, exploring the perspectives of implementation partners in greater depth and considering the role of contextual factors in shaping implementation outcomes could provide valuable insights for future intervention design and implementation efforts.

### Conclusions

This paper examined factors influencing the implementation of the digital PA intervention “Active Lives” from the perspectives of the core implementation team and partners. Key issues include complexities with the identification of influencers and decision makers, navigating approval processes within tight time frames and competing demands. Misunderstandings of Active Lives and differing views on suitable interventions for older adults alongside loyalty to existing interventions emerged as barriers. Even partners eager to implement Active Lives tended to choose low-reach and effortful approaches, but a more directive strategy with anecdotal evidence helped promote passive, high-impact routes.

## Supplementary material

10.2196/64953 Multimedia Appendix 1 Implementation team topic guide.

10.2196/64953 Multimedia Appendix 2 Implementation partner topic guide.

10.2196/64953 Checklist 1 StaRI (Standards for Reporting Implementation Studies) checklist.

10.2196/64953 Checklist 2 COREQ (Consolidated Criteria for Reporting Qualitative Research) checklist.

## Abbreviations

AHSN: Academic Health Sciences Network
COREQ: Consolidated Criteria for Reporting Qualitative Research
HCP: health care professional
NHS: National Health Service
PA: physical activity
RCT: randomized controlled trial
StaRI: Standard for Reporting Implementation Studies
