# Supplementary material for: Implementing a Digital Physical Activity Intervention for Older Adults: Qualitative Study
Source: JMIR Aging. 2025 Aug 21;8:e64953. doi: 10.2196/64953 (PMC12370260; doi:10.2196/64953)
Supplement: Multimedia Appendix 1 [file aging-v8-e64953-s001.docx]

**Interview Schedule: Implementation Team**

First of all, can you tell me about your role in ‘IDA’, the *Active Lives* roll-out project?

How have you been getting on with getting people and organisations to implement *Active Lives*?

Can you tell me about something that has gone well with spreading/implementing?
(Can you tell me about whether this route is still working?)

Can you talk me though what’s been tricky with the rollout?
(Probe for multiple issues. Other ways to ask: Can you tell me about anything else has been a problem, difficulty, hurdle, barrier)

Tell me about where things just haven’t got off the ground?
(Probe for multiple examples)

Can you talk me through something that didn’t go very well?
(Probe for multiple examples)

Can you tell me about how *Active Lives* seemed to fit with the needs and priorities of the people and organisations you’ve been in touch with? (probe alignment/fit and lack of alignment/fit)

I wonder about whether *Active Lives* seemed workable for them? (probe workable and unworkable)

What about how *Active Lives* fit with how they usually do things? (probe fit with usual practice and lack of it)

What tasks did the people you’ve been in touch with need to do to get *Active Lives* going?

How did people go about it?

What activities or discussions did you hear about?

When trying to roll out *Active Lives* what did people want or need from the research team?

So far, what strikes you as being important in implementing *Active Lives?*

(probe for multiple issues. Other ways to ask: what else appeared to be important? What else seems to be influential? What other considerations are there? What else matters?)

I wondering if there is anything that isn’t seeming that important, that you had thought might be?
(probe for multiple issues)

Can you tell me about anything that has surprised you when trying to spread *Active Lives?*

Can you tell me about anything you’ve learned about getting an intervention like *Active Lives* adopted?

How has this influenced your ongoing efforts to roll out *Active Lives*?
(Other way to ask: Can you tell me about any changes you are making to the way you do things? )

Finally, is there anything else you’d like to tell me about?

--END--

| **General open/ended prompts**   - How did you find that? - What was that like? - Can you tell me more about x? - I’d love to hear a bit more about what you were saying about X? - Can you expand on x? - Would you be able to give me an example of X? - What did you mean by x? |
| --- |
| **Prompts to find out more about specific situations/examples**   - Can you tell me more about what happened there? - Can you talk me through what happened/what happened next? - What do you think was going on? - How did you make sense of that situation? - How did you handle that? |
