# Supplementary material for: Implementing a Digital Physical Activity Intervention for Older Adults: Qualitative Study
Source: JMIR Aging. 2025 Aug 21;8:e64953. doi: 10.2196/64953 (PMC12370260; doi:10.2196/64953)
Supplement: Multimedia Appendix 2 [file aging-v8-e64953-s002.docx]

**Interview Schedule: Implementation Partners**

[The topics probed about varied slightly depending on the role of the interviewee, and whether the organisation did or did not implement Active Lives]

1. Can you tell me a bit about your role?
2. Can you tell me about any ways in which your service is currently involved in helping older adults to increase physical activity (probe: What strategies have you used previously to increase exercise amongst older adults?)
3. How did you first hear about Active Lives?
4. How did you become involved in the implementation of Active Lives?
5. Can you talk me through your role and the journey from first hearing about active lives to your most present encounter?
6. When you first heard of it, how did you think Active Lives was going to fit into your current work? Certain project, campaigns, agendas?
7. How does Active Lives differ from previous strategies?
8. How do you think Active Lives differs from other products/services aimed at increasing PA in older adults? Can you tell me about any overlap?
9. What do you think might be important to implementing organisation when reviewing something like Active Lives?
10. Did you have a vision of what you wanted to achieve with Active Lives? *Has that changed over time? Why?*
11. Did you have a clear vision of how Active Lives was going to fit in with your work? *Has that changed over time? Why?*
12. Who were the main stakeholders involved in the decision making of whether and how you would use Active Lives in your service?

**Use these questions below if you know that the interviewee has managed to implement Active Lives in their service to some degree:**

1. So I understand that you’ve been trying to implement Active Lives into your service. Can you talk me through the approach that you’ve taken? (probe why this approach).
2. Can you tell me about anything that has worked well in this approach?
3. Can you talk me through anything that hasn’t worked so well?
4. How did the process of trying to implement Active Lives into your service feel overall?
5. How is the website promoted?
6. Who is responsible for promotion?
7. What do you feel have been the main successes in implementing Active Lives?
8. Can you tell me about any obstacles you’ve encountered? *How were they managed?*
9. In hindsight, is there anything you would have done differently?
10. Is there anything else you’d like to share?

**Use these questions below if you know that the interviewee has NOT managed to implement Active Lives into their service:**

1. So I understand that you weren’t able to implement Active Lives into your service in the end, would you mind telling me a bit about what happened? (probe: how was decision made, who was involved?).
2. What would you say were the main barriers to using Active Lives in your service?
3. what do you think would have needed to be different for you to have implemented Active Lives into your service?
4. Is there anything else you’d like to share?

| **General open/ended prompts**   - How did you find that? - What was that like? - Can you tell me more about x? - I’d love to hear a bit more about what you were saying about X? - Can you expand on x? - Would you be able to give me an example of X? - What did you mean by x? |
| --- |
| **Prompts to find out more about specific situations/examples**   - Can you tell me more about what happened there? - Can you talk me through what happened/what happened next? - What do you think was going on? - How did you make sense of that situation? - How did you handle that? |

**Interview Schedule: Implementation Team**

First of all, can you tell me about your role in ‘IDA’, the *Active Lives* roll-out project?

How have you been getting on with getting people and organisations to implement *Active Lives*?

Can you tell me about something that has gone well with spreading/implementing?
(Can you tell me about whether this route is still working?)

Can you talk me though what’s been tricky with the rollout?
(Probe for multiple issues. Other ways to ask: Can you tell me about anything else has been a problem, difficulty, hurdle, barrier)

Tell me about where things just haven’t got off the ground?
(Probe for multiple examples)

Can you talk me through something that didn’t go very well?
(Probe for multiple examples)

Can you tell me about how *Active Lives* seemed to fit with the needs and priorities of the people and organisations you’ve been in touch with? (probe alignment/fit and lack of alignment/fit)

I wonder about whether *Active Lives* seemed workable for them? (probe workable and unworkable)

What about how *Active Lives* fit with how they usually do things? (probe fit with usual practice and lack of it)

What tasks did the people you’ve been in touch with need to do to get *Active Lives* going?

How did people go about it?

What activities or discussions did you hear about?

When trying to roll out *Active Lives* what did people want or need from the research team?

So far, what strikes you as being important in implementing *Active Lives?*

(probe for multiple issues. Other ways to ask: what else appeared to be important? What else seems to be influential? What other considerations are there? What else matters?)

I wondering if there is anything that isn’t seeming that important, that you had thought might be?
(probe for multiple issues)

Can you tell me about anything that has surprised you when trying to spread *Active Lives?*

Can you tell me about anything you’ve learned about getting an intervention like *Active Lives* adopted?

How has this influenced your ongoing efforts to roll out *Active Lives*?
(Other way to ask: Can you tell me about any changes you are making to the way you do things? )

Finally, is there anything else you’d like to tell me about?

--END--

| **General open/ended prompts**   - How did you find that? - What was that like? - Can you tell me more about x? - I’d love to hear a bit more about what you were saying about X? - Can you expand on x? - Would you be able to give me an example of X? - What did you mean by x? |
| --- |
| **Prompts to find out more about specific situations/examples**   - Can you tell me more about what happened there? - Can you talk me through what happened/what happened next? - What do you think was going on? - How did you make sense of that situation? - How did you handle that? |
