# Supplementary material for: Implementing a Digital Physical Activity Intervention for Older Adults: Qualitative Study
Source: JMIR Aging. 2025 Aug 21;8:e64953. doi: 10.2196/64953 (PMC12370260; doi:10.2196/64953)
Supplement: Checklist 1 [file aging-v8-e64953-s003.docx]

| Item | | Implementation Strategy | Page | Intervention | Page |
| --- | --- | --- | --- | --- | --- |
| **Title and Abstract** | | | | | |
| Title | 1 | Identification as an implementation study, and description of the methodology in the title and/or keywords | | | Title |
| Abstract | 2 | Identification as an implementation study, including a description of the implementation strategy to be tested, the evidence- based intervention being implemented, and defining the key implementation and health outcomes. | | | Abstract |
| **Introduction** | | | | | |
| Introduction | 3 | Description of the problem, challenge or deficiency in healthcare or public health that the intervention being implemented aims to address. | | | Introduction |
| Rationale | 4 | The scientific background and rationale for the implementation strategy (including any underpinning theory/framework/model, how it is expected to achieve its effects and any pilot work). | Introduction and table 1 | The scientific background and rationale for the intervention being implemented (including evidence about its effectiveness and how it is expected to achieve its effects). | Introduction |
| Aims and Objectives | 5 | The aims of the study, differentiating between implementation objectives and any intervention objectives. | | | Introduction |
| **Methods: description** | | | | | |
| Design | 6 | The design and key features of the evaluation, (cross referencing to any appropriate methodology reporting standards) and any changes to study protocol, with reasons | | | Methods- research design, ethics and reporting |
| Context | 7 | The context in which the intervention was implemented. (Consider social, economic, policy, healthcare, organisational barriers and facilitators that might influence implementation elsewhere). | | | Methods- implementation strategy and context |
| Targeted ‘sites’ | 8 | The characteristics of the targeted ‘site(s)’ (e.g locations/personnel/resources etc.) for implementation and any eligibility criteria. | Methods- implementation strategy and context | The population targeted by the intervention and any eligibility criteria. | Introduction;  Methods- implementation strategy and context |
| Description | 9 | A description of the implementation strategy | Methods- implementation strategy and context | A description of the intervention | Introduction |
| Sub-groups | 10 | Any sub-groups recruited for additional research tasks, and/or nested studies are described | | | Introduction (describes AL as a nested study within a larger study)  Methods- implementation strategy and context (describes the IPs as separate from the target pop for the intervention, but recruited for implementation evaluation) |
| **Methods: evaluation** | | | | | |
| Outcomes | 11 | Defined pre-specified primary and other outcome(s) of the implementation strategy, and how they were  assessed. Document any pre-determined targets | Introduction (aims)  Methods- data collection | Defined pre-specified primary and other outcome(s) of the intervention (if assessed), and how they were assessed. Document any pre-determined targets | Introduction  (Cross referenced to RCT results and ongoing evaluation as part of larger intervention) |
| Process evaluation | 12 | Process evaluation objectives and outcomes related to the mechanism by which the strategy is expected to work | | | Methods |
| Economic evaluation | 13 | Methods for resource use, costs, economic outcomes and analysis for the implementation strategy | n/a | Methods for resource use, costs, economic outcomes and analysis for the intervention | n/a |
| Sample size | 14 | Rationale for sample sizes (including sample size calculations, budgetary constraints, practical considerations, data saturation, as appropriate) | | | Methods- data collection |
| Analysis | 15 | Methods of analysis (with reasons for that choice) | | | Methods – data analysis |
| Sub-group analyses | 16 | Any a priori sub-group analyses (e.g. between different sites in a multicentre study, different clinical or demographic populations), and sub-groups recruited to specific nested research tasks | | | N/A |
| **Results** | | | | | |
| Characteristics | 17 | Proportion recruited and characteristics of the recipient population for the implementation strategy | Method- data collection  Findings- participants | Proportion recruited and characteristics (if appropriate) of the recipient population for the intervention | N/A |
| Outcomes | 18 | Primary and other outcome(s) of the implementation strategy | Findings  Discussion- key findings and implications | Primary and other outcome(s) of the Intervention (if assessed) | N/A |
| Process outcomes | 19 | Process data related to the implementation strategy mapped to the mechanism by which the strategy is expected to work | | | N/A – |
| Economic evaluation | 20 | Resource use, costs, economic outcomes and analysis for the implementation strategy | N/A | Resource use, costs, economic outcomes and analysis for the intervention | N/A |
| Sub-group analyses | 21 | Representativeness and outcomes of subgroups including those recruited to specific research tasks | | | Methods- data collection. Findings – participants, |
| Fidelity/ adaptation | 22 | Fidelity to implementation strategy as planned and adaptation to suit context and preferences | Method- implementation strategy and context. Findings.  Discussion- key findings and implications | Fidelity to delivering the core components of intervention (where measured) | N/A |
| Contextual changes | 23 | Contextual changes (if any) which may have affected outcomes | | | Findings |
| Harms | 24 | All important harms or unintended effects in each group | | | N/A |
| **Discussion** | | | | | |
| Structured discussion | 25 | Summary of findings, strengths and limitations, comparisons with other studies, conclusions and implications | | | Discussion |
| Implications | 26 | Discussion of policy, practice and/or research implications of the implementation strategy (specifically including scalability) | Discussion- key findings and implications | Discussion of policy, practice and/or research implications of the intervention (specifically including sustainability) | Introduction. Discussion- key findings and implications |
| **General** | | | | | |
| Statements | 27 | Include statement(s) on regulatory approvals (including, as appropriate, ethical approval, confidential use of routine data, governance approval), trial/study registration (availability of protocol), funding and conflicts of interest | | | Introduction (ISRCTN#).  Methods (ethics approval) |
