# Supplementary material for: Implementing a Digital Physical Activity Intervention for Older Adults: Qualitative Study
Source: JMIR Aging. 2025 Aug 21;8:e64953. doi: 10.2196/64953 (PMC12370260; doi:10.2196/64953)
Supplement: Checklist 2 [file aging-v8-e64953-s004.docx]

|  | | Item | Guide questions/description | Manuscript section where information can be found |
| --- | --- | --- | --- | --- |
| **Domain 1: Research team and reflexivity** | | | | |
| Personal Characteristics | 1 | Interviewer/facilitator | Which author/s conducted the interview or focus group? | Method- data collection |
|  | 2 | Credentials | What was their occupation at the time of the study? | Method- data collection |
|  | 3 | Occupation | What was their occupation at the time of the study? | Method- data collection |
|  | 4 | Gender | Was the researcher male or female? | Method- data collection |
|  | 5 | Experience and training | What experience or training did the researcher have? | Method- data collection |
| Relationship with participants | 6 | Relationship established | Was a relationship established prior to study commencement? | Method- data collection |
|  | 7 | Participant knowledge of the interviewer | What did the participants know about the researcher? e*.g. personal goals, reasons for doing the research* | Method- reflexivity |
|  | 8 | Interviewer characteristics | What characteristics were reported about the interviewer/facilitator? e.g. *Bias, assumptions, reasons and interests in the research topic* | Method- data collection, reflexivity  Discussion – study strengths and limitations |
| **Domain 2: study design** | | | | |
| Theoretical framework | 9 | Methodological orientation and Theory | What methodological orientation was stated to underpin the study? *e.g. grounded theory, discourse analysis, ethnography, phenomenology, content analysis* | Method- research design, ethics and reporting; data analysis |
| Participant selection | 10 | Sampling | How were participants selected? *e.g. purposive, convenience, consecutive, snowball* | Method- data collection |
|  | 11 | Method of approach | How were participants approached? e*.g. face-to-face, telephone, mail, email* | Method- data collection |
|  | 12 | Sample size | How many participants were in the study? | Findings- participants |
|  | 13 | Non-participation | How many people refused to participate or dropped out? Reasons? | Method- data collection  Discussion.  Our recruitment method does not allow us to know why participants did not respond to our invitation to participate. |
|  | 14. | Setting of data collection | Where was the data collected? e*.g. home, clinic, workplace* | Method- data collection |
|  | 15. | Presence of non-participants | Was anyone else present besides the participants and researchers? | Method- data collection |
|  | 16. | Description of sample | What are the important characteristics of the sample? *e.g. demographic data, date* | Findings- participants |
| Data collection | 17. | Interview guide | Were questions, prompts, guides provided by the authors? Was it pilot tested? | Method- data collection  Supplementary Materials 3 |
|  | 18. | Repeat interviews | Were repeat interviews carried out? If yes, how many? | Findings- participants |
|  | 19. | Audio/visual recording | Did the research use audio or visual recording to collect the data? | Method- data collection |
|  | 20 | Field notes | Were field notes made during and/or after the interview or focus group? | Methods- data collection |
|  | 21. | Duration | What was the duration of the interviews or focus group? | Methods- data collection |
|  | 22. | Data saturation | Was data saturation discussed? | Not reported in main manuscript for conciseness.  The authors are very cautious about claims of data saturation in thematic analysis (see, for example, arguments in Braun & Clarke, 2021; “To Saturate or not to saturate” Qual Res in Sport, Exercise Health).  Recruitment was constrained by participant response rate and time limitations of the project. |
|  | 23. | Transcripts returned | Were transcripts returned to participants for comment and/or correction? | n/a  (member checks with participants were not conducted, professional transcribers transcribed the interviews and researchers checked for accuracy) |
| **Domain 3: analysis and findings** | | | | |
| Data analysis | 24. | Number of data coders | How many data coders coded the data? | Method- data analysis |
|  | 25. | Description of the coding tree | Did authors provide a description of the coding tree? | n/a (coding tree not used within reflexive thematic analysis) |
|  | 26. | Derivation of themes | Were themes identified in advance or derived from the data? | Method- data analysis |
|  | 27. | Software | What software, if applicable, was used to manage the data? | Method- data analysis |
|  | 28. | Participant checking | Did participants provide feedback on the findings? | n/a  (member checks with implementation partners were not conducted, but the implementation team participants were also involved in validation of the analysis) |
| Reporting | 29. | Quotations presented | Were participant quotations presented to illustrate the themes / findings? Was each quotation identified? e*.g. participant number* | Findings |
|  | 30. | Data and findings consistent | Was there consistency between the data presented and the findings? | Findings |
|  | 31. | Clarity of major themes | Were major themes clearly presented in the findings? | Findings; Table 2 |
|  | 32. | Clarity of minor themes | Is there a description of diverse cases or discussion of minor themes? | Findings; Table 3 |
